# A single nucleotide polymorphism of AIRE gene located in the 21q22.3 increases the risk of rheumatoid arthritis

**DOI:** 10.18632/oncotarget.17746

**Published:** 2017-05-10

**Authors:** Yuan-Sheng Xu, Xi-Jia Jiang, Jian-Min Chen

**Affiliations:** ^1^ Department of Orthopaedics, Bayi Hospital Affiliated to Nanjing University of Chinese Medicine, Nanjing 210002, China; ^2^ Department of Orthopaedics, Changzhou No.2 People's Hospital, Changzhou 213003, China

**Keywords:** AIRE, single nucleotide polymorphism, rheumatoid arthritis

## Abstract

Several studies addressed the association of autoimmune regulator (AIRE) gene polymorphism with the risk of rheumatoid arthritis (RA); however, their conclusions were inconsistent. For better investigating the effects of this polymorphism on the risk of RA, we conducted this study to evaluate the role of AIRE rs2075786 polymorphism in the risk of RA. Four eligible studies involving 6,755 cases and 7,970 controls were identified by searching the databases of PubMed, CNKI and EMBASE up to February 2017. Our study revealed that AIRE rs2075786 polymorphism was associated with an increased risk of RA under all genetic models. In the subgroup analysis, AIRE rs2075786 polymorphism contributed to RA susceptibility among Asians, but not among Caucasians. To summarize,, this meta-analysis confirms that AIRE rs2075786 polymorphism may play a significant role in increasing the risk of RA. Stratification analysis by ethnicity reveals that AIRE rs2075786 polymorphism is associated with an increased risk of RA among Asians, but not among Caucasians. These findings need further validation in the large multicenter case-control studies.

## INTRODUCTION

Rheumatoid arthritis (RA), is a chronic inflammatory autoimmune disease in which hyperplasia, hypertrophy and angiogenesis of synovial tissue contribute to inflammatory joint destruction [1]. In industrialised countries, RA affects 0.5–1.0% of adults, with 5–50 per 100 000 new cases annually [2, 3]. 50% of the risk for development of RA is attributable to genetic factors [4]. Moreover, the role of common genetic variation in determining the range of individual susceptibility within the population is increasingly recognized [5].

The autoimmune regulator (AIRE) gene is located in the 21q22.3 region, and is 12.5 kb in length, and contains 14 exons that encode a 545 amino acid protein of 58 kD [6, 7]. AIRE plays a key role in shaping central immunological tolerance by facilitating negative selection of T cells in the thymus, building the thymic microarchitecture, and inducing a specific subset of regulatory T cells [8]. Moreover, many studies demonstrated that thymus of AIRE knockout mice appears normal in terms of thymocytes number, but AIRE knockout mice and autoimmune polyendocrinopaythy-candidiasis-ectodermal dystrophy (APECED) patients, both exhibits autoimmune disease [9–11]. Liu *et al.* found significantly lower levels of AIRE expression were associated with the development of thymoma-related autoimmune diseases [12]. Furthermore, Terao *et al.* found that downregulation of AIRE by genetic polymorphisms may trigger auto-inflammation in RA [13].

Over 100 mutations in the AIRE gene have been reported in the Human Gene Mutation Database, varying from single nucleotide substitutions to large deletions spread out across the coding sequence. AIRE gene polymorphism has been studied in various autoimmune disease, such as Graves’ disease [14], type 1 diabetic [15] and alopecia areata [16]. A GWAS study identified the strong association between rs2075786 polymorphism and RA risk. However, the association between AIRE rs2075786 polymorphism and RA risk remains obscure because of contradictory and inconclusive findings of other published studies. Thus, we undertook this meta-analysis to evaluate whether AIRE rs2075786 polymorphism was associated with RA risk.

## RESULTS

### Study characteristics

**Figure 1:**
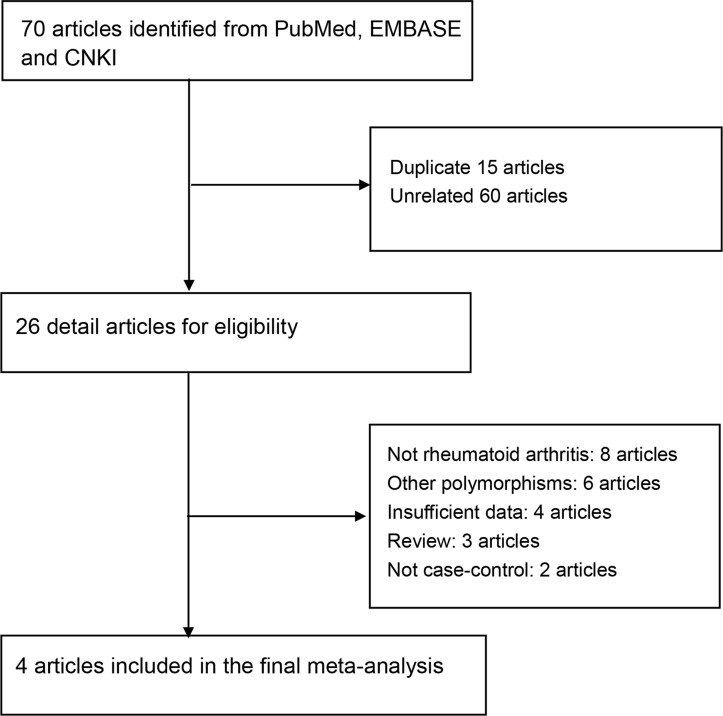
Selection for eligible citations included in this meta-analysis

70 articles were retrieved after initial search. The selection process of eligible articles is shown in Figure 1. 44 articles were excluded; of which 15 were duplicates and 29 were unrelated to the topic. Among of the remaining 26 articles, 14 articles did not investigate the association between AIRE rs2075786 polymorphism and RA risk; one article was not a case-control study; 4 articles did not have sufficient genotype frequencies; 3 articles were reviews. Finally, 4 studies were available for our topic in this meta-analysis, consisting of 6,755 cases and 7,970 controls. The detailed characteristics of the included studies are listed in Table 1.

**Table 1:** Characteristics of included studies for the association between rs751402 and rheumatoid arthritis risk

Author	year	Country	Ethnicity	SOC	Genotype method	Case			Control			HWE	NOS
						CC	CT	TT	CC	CT	TT		
Feng	2015	China	Asian	HB	TaqMan	173	350	168	273	364	163	0.013	6
Garcia-Lozano	2013	Spain	Caucasian	HB	TaqMan	364	95	6	371	95	6	0.977	6
Shao	2013	China	Asian	HB	SNaPshot assay	61	116	55	105	115	53	0.038	7
Terao	2011	Japan	Asian	HB	TaqMan	2091	2502	774	2811	2857	757	0.450	8

SOC, source of controls; PB, population-based controls; HB, hospital-based controls; HWE, Hardy–Weinberg Equilibrium; NOS, Newcastle-Ottawa Scale.

### Quantitative synthesis of data

**Figure 2:**
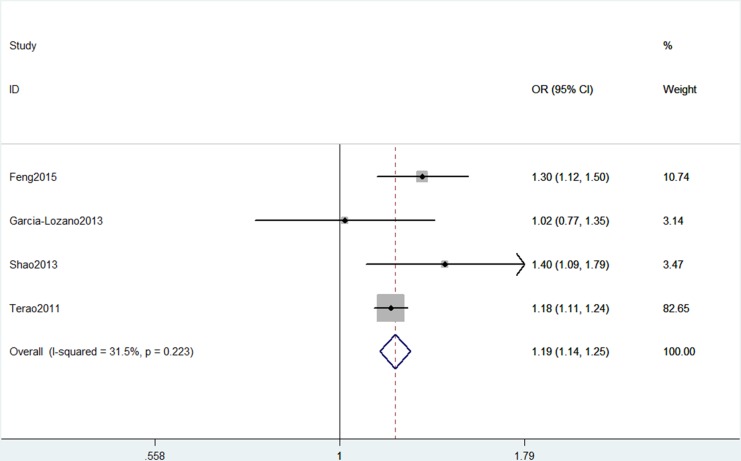
Forest plot shows odds ratio for the association between AIRE gene rs2075786 polymorphism and RA risk (A vs. G)

**Figure 3:**
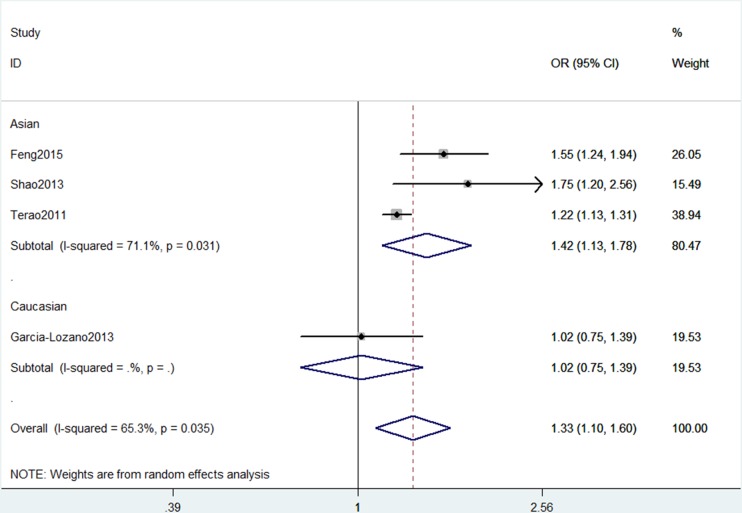
Stratification analyses of ethnicity shows odds ratio for the association between AIRE gene rs2075786 polymorphism and RA risk (AA+GA vs. GG)

In the overall analysis, AIRE rs2075786 polymorphism showed positive correlation with RA risk and could be viewed as a risk factor of RA under five models (Table 2). In the allele model, individuals carrying the AA genotype had a 19% higher risk of RA compared to GG genotype carriers (A vs. G: OR, 1.19; 95% CI, 1.14–1.25, *P* < 0.001, Figure 2). Subgroup analysis by ethnicity revealed that AIRE rs2075786 polymorphism could contribute to the risk of RA among Asians (Table 3), but not among Caucasians (AA+GA vs. GG: OR, 1.42; 95% CI, 1.13–1.78, *P* = 0.002, Figure 3). When stratified by Hardy-Weinberg equilibrium (HWE) status, significant associations were also observed between AIRE rs2075786 polymorphism and RA risk (Table 3).

**Table 2:** Analysis for the effect of rs2075786 on the risk of rheumatoid arthritis

Genetic model	Statistics		Heterogeneity		Publication bias	
	OR(95%CI)	*P*	*P*_heterogeneity_	I^2^(%)	*P*begg	*P*Egger
Allele (A vs. G)	**1.19 (1.14,1.25)**	< 0.001	0.223	31.5	0.497	0.664
Dominant (AA+GA vs. GG)	**1.33 (1.10,1.60)**	0.004	0.035	65.3	0.497	0.517
Recessive (AA vs. GA+GG)	**1.26 (1.15,1.39)**	< 0.001	0.985	0.0	0.497	0.419
Homozygous (AA vs. GG)	**1.42 (1.28,1.57)**	< 0.001	0.508	0.0	1.000	0.601
Heterozygous (GA vs. GG)	**1.29 (1.07,1.57)**	0.009	0.046	62.5	0.497	0.448

*Bold values are statistically significant (*P* < 0.05).

**Table 3:** Summary of the subgroup analyses in this meta-analysis

Comparison	Category	Category	Studies	OR (95% CI)	*P*-value	*P* for heterogeneity
A vs. G	Ethnicity	Asian	3	**1.20 (1.14,1.26)**	< 0.001	0.206
		Caucasian	1	1.02 (0.77,1.35)	0.908	N/A
	HWE	No	2	**1.32 (1.17,1.50)**	< 0.001	0.626
		Yes	2	**1.17 (1.11,1.23)**	< 0.001	0.327
AA+GA vs. GG	Ethnicity	Asian	3	**1.42 (1.13,1.78)**	0.002	0.031
		Caucasian	1	1.02 (0.75,1.39)	0.905	N/A
	HWE	No	2	**1.60 (1.32,1.94)**	< 0.001	0.590
		Yes	2	**1.19 (1.06,1.34)**	0.003	0.274
AA vs.GA+ GG	Ethnicity	Asian	3	**1.26 (1.15,1.39)**	< 0.001	0.994
		Caucasian	1	1.02 (0.33,3.17)	0.979	N/A
	HWE	No	2	**1.26 (1.02,1.56)**	0.031	0.914
		Yes	2	1.26 (1.13,1.40)	< 0.001	0.710
AA vs. GG	Ethnicity	Asian	3	**1.42 (1.28,1.58)**	< 0.001	0.368
		Caucasian	1	1.02 (0.33,3.19)	0.974	N/A
	HWE	No	2	1.67 (1.30,2.13)	< 0.001	0.747
		Yes	2	1.37 (1.22,1.54)	< 0.001	0.609
GA vs. GG	Ethnicity	Asian	3	**1.38 (1.09,1.76)**	0.008	0.033
		Caucasian	1	1.02 (0.74,1.40)	0.907	N/A
	HWE	No	2	**1.57 (1.28,1.93)**	< 0.001	0.577
		Yes	2	1.17 (1.08,1.26)	< 0.001	0.390

SOC, source of controls; PB, population-based controls; HB, hospital-based controls; HWE: Hardy–Weinberg equilibrium.

**Figure 4:**
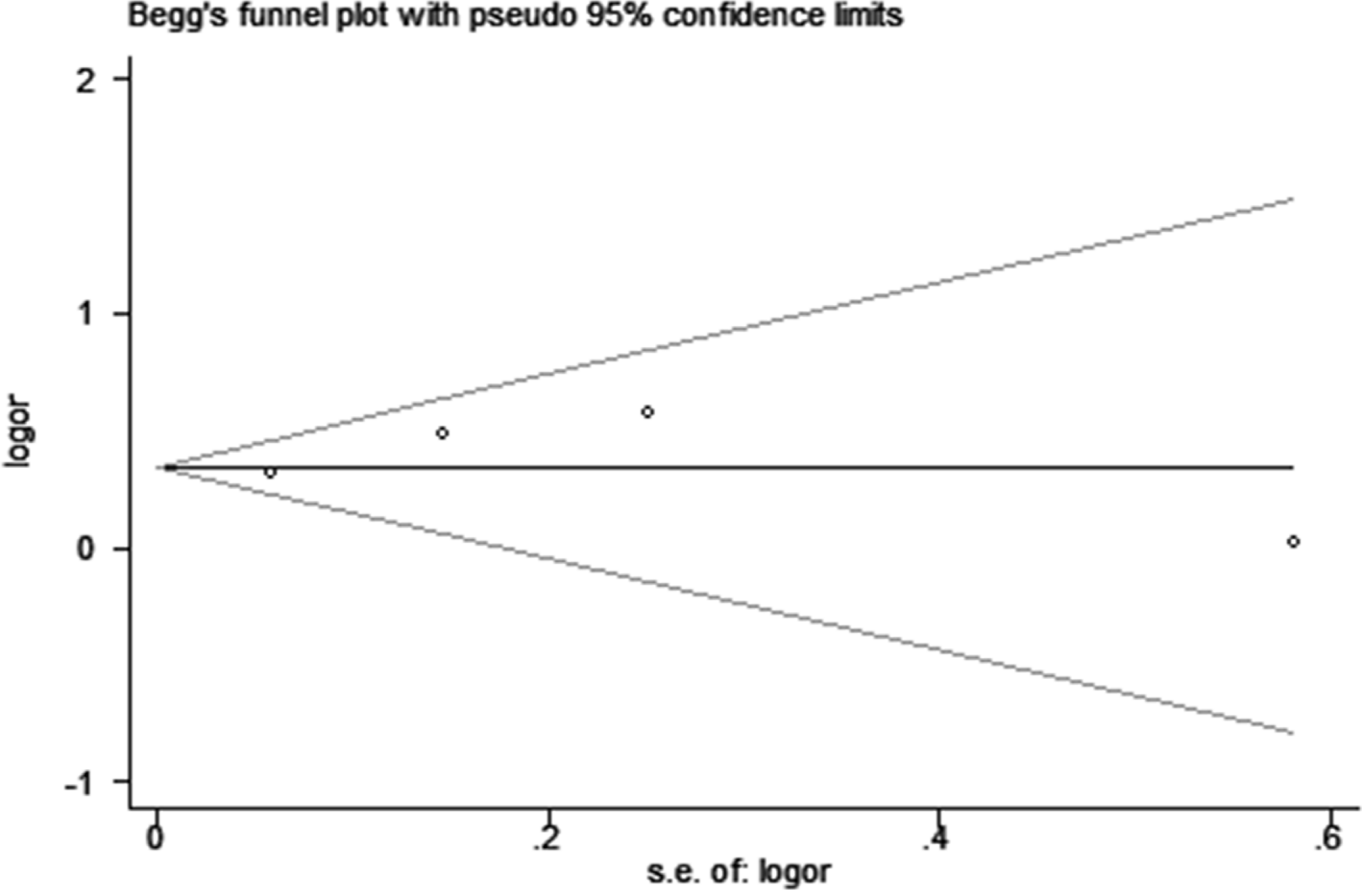
Begg's tests between AIRE gene rs2075786 polymorphism and RA risk (AA vs. GG)

Sensitivity analysis using the leave-one-out cross-validation method was conducted to assess the impact of each single study on the overall risk estimates. The omission of each individual study did not have substantial influence on the risk estimates, supporting the credibility and reliability of this meta-analysis. Publication bias was assessed by Begg's funnel plot and quantitative Egger's test (Table 2). We did not find any obvious publication bias for the association between AIRE rs2075786 polymorphism and RA risk (AA vs. GG, Figure 4)

## DISCUSSION

AIRE is a transcriptional regulator primarily expressed in medullary thymic epithelial cells, and plays a functional role in thymocyte education and negative selection by controlling the expression of peripheral antigens in thymus [17]. AIRE in peripheral organs may provide hints to the involvement of AIRE in the predisposition or progression in RA [13]. According to the GEO database, the risk allele of rs2075876 polymorphism decreased the transcription level of AIRE. A series of studies started to investigate the role of AIRE rs2075786 polymorphism in the risk of RA, but with inconsistent results.

A GWAS study conducted by Terao *et al.* found that AIRE gene rs2075786 polymorphism increased the risk of RA among the Japanese population [13]. The significant association was also observed in the subsequent studies among Chinese Han populations [18, 19]. However, Garcia-Lozano *et al.* failed to replicate this association in a Caucasian population [20]. Our meta-analysis suggested that AIRE rs2075786 polymorphism could contribute to the risk of RA, especially among Asians. The results were consistent with the included studies from Asia. A possible explanation for the difference between Asians and Caucasians is that AIRE rs2075786 polymorphism may have an ethnicity-specific effect. According to 1000 Genomes Browser, A allele frequency in China's Beijing population is 0.8155, while in the Spanish population of 0.2710. Our results found that the A allele frequency was 0.3715 in Asians, which was higher than that of Caucasians (0.1142). Except for different distribution of gene functional polymorphisms among different races, we thought the following reasons including small sample size of single study, clinical heterogeneity, different environments, or uncorrected multiple hypothesis testing may explain the inconsistent findings between Caucasian populations and Asian populations. It is noteworthy that the sample sizes of Caucasians and Asians were limited in this meta-analysis. Therefore, we should interpret the data with caution.

High heterogeneities were observed in the dominant (*P*_heterogeneity_ = 0.035, I^2^ = 65.3) and heterozygous models (*P*_heterogeneity_ = 0.046, I^2^ = 62.5), which may have effects on our results. Subgroup analyses and sensitivity analysis were conducted to uncover the source of heterogeneity. Due to limited studies in this meta-analysis, we cannot undertake the meta-regression. Unfortunately, we did not detect ethnicity and HWE status were account for high heterogeneity. However, sensitivity analysis indicated our data were stable and robust.

Although we found the positive finding, the limitations of this meta-analysis should be taken into careful consideration. First, RA is a multifactorial disease, and thus the function of single SNP is limited. Second, the sample sizes of some individual studies were very small, which could deviate from the truly results. Third, it is worth noting that this meta-analysis only contains one studies about Caucasians, and the result should be interpreted with caution. Fourth, only four studies were included in this meta-analysis. Fifth, the heterogeneity of some genetic models in this meta-analysis is high. Sixth, genotyping methods were different in some studies, which will affect the bias. Finally, no further subgroup analyses such as smoking and age were conducted due to the lack of eligible data.

In summary, AIRE rs275786 polymorphism was associated with the increased risk of RA, which may be viewed as a new biomarker to early screening and prevention of RA

## MATERIALS AND METHODS

### Search strategy and selection criteria

Two investigators carried out a systematic electronic search independently in PubMed, EMBASE and China Knowledge Resource Integrated Database to identify relevant studies. The following key search terms: “autoimmune regulator,” “AIRE,” “APECED,” “polymorphism,” “single nucleotide polymorphism,” “SNP” “rheumatoid arthritis”. No restrictions were placed on the search. Additional initially omitted studies (such as reference lists of identified studies) have been identified by hand screening.

Eligible studies conformed to the following criteria: (1) investigating the relationship between AIRE rs2075786 polymorphism and risk of RA; (2) sufficient data for calculating the pooled odds ratio (ORs) with 95% confidence interval (CI); (3) case-control studies; (4) studied on human beings. Exclusion criteria were as follows: (1) duplicate studies; (2) lack of enough genotype data or data for calculating genotype distribution; (3) not case-control studies; (4) case only studies.

### Data extraction and quality assessment

Two investigators reviewed and extracted data independently in accordance with the inclusion criteria. From each study, the following information was extracted: name of first author, publication year, country of origin, ethnicity, numbers of cases and controls, and cancer type. When studies included subjects of more than one ethnicity, genotype data were extracted separately. The Newcastle-Ottawa Scales (NOS) were used to assess the quality of the selected studies [21]. The discrepancies were resolved by discussion or consulting with a third reviewer.

### Statistical analysis

All statistical analyses were performed using the Stata 11.0 software (StataCorp, College Station, TX, USA). Pooled ORs with corresponding 95% CIs were calculated to evaluate the strength of relationship between AIRE rs2075786 polymorphism and risk of RA. Stratification analyses were carried out by ethnicity and HWE. *P* < 0.05 was considered statistically significant. Taking possible between-study heterogeneity into consideration, we considered the presence of significant heterogeneity at the 10% level of significance and values of I^2^ exceeding 50% as an indicator of significant heterogeneity. When no heterogeneity was found with *P* > 0.10 or I^2^ < 50%, a fixed-effect model was used. Otherwise, a random-effects model was applied [22]. Sensitivity analysis was conducted to determine the effect on the test of heterogeneity and evaluate the stability of the results by omitting each study in turn. Genotype distributions in the controls were tested for confirmation of HWE using the χ^2^ test. Publication bias was evaluated by visual inspection of symmetry of Begg's funnel plot and assessment of Egger's test [23]; *P* < 0.05 was regarded as representative of statistical significance.
